# First Record of an Invasive Fruit Fly Belonging to *Bactrocera dorsalis* Complex (Diptera: Tephritidae) in Europe

**DOI:** 10.3390/insects9040182

**Published:** 2018-12-03

**Authors:** Francesco Nugnes, Elia Russo, Gennaro Viggiani, Umberto Bernardo

**Affiliations:** 1 CNR, Institute for Sustainable Plant Protection, 80055 Portici, Italy; francesco.nugnes@ipsp.cnr.it (F.N.); elia.russo@ipsp.cnr.it (E.R.); 2 Department of Agriculture, University of Naples “Federico II”, 80055 Portici, Italy; genviggi@unina.it

**Keywords:** *Bactrocera dorsalis*, *B. kandiensis*, first record, fruit fly, invasive species, male lure, species complex

## Abstract

Emerging pests are increasingly threatening fruit orchard health across the Mediterranean area. Tephritidae, representing serious threats for Europe, are numerous, and the fruit flies *Bactrocera zonata* and those belonging to *Bactrocera dorsalis* complex are among the most alarming species. These species are highly polyphagous and *B. zonata* has already spread to some Mediterranean countries. Due to these ongoing threats, in the Campania Region (southern Italy), a survey with traps and infested fruits analysis was performed with the aim of detecting the presence of species of *Bactrocera dorsalis* complex. In two mixed fruit-trees fields, some adults belonging to a species of *Bactrocera* were captured in traps baited with the highly attractive male lure (methyl eugenol). They were distinguished from similar-looking *Bactrocera* spp. by morphological and molecular comparative analyses. Considering the existing morphological keys, specimens were tentatively identified as *B. dorsalis* but molecular characterization with COI split them into two clades. Some specimens were grouped with *B. dorsalis* similar to *B. kandiensis* and *B. kandiensis* and others in a clade including *B. dorsalis* and *B. invadens* (syn. *B. dorsalis*). ITS1 sequences instead confirmed morphological identification. The integrative approach allowed identifying all the specimens collected as belonging to the *B. dorsalis* complex. This finding represents the first field interception in Europe of a member of one of the most dangerous groups of fruit flies.

## 1. Introduction

Emerging pests are increasingly threatening fruit orchard health across the Mediterranean area. The dynamics of these threats are often driven by climate changes that not only weaken trees, increasing their vulnerability, but also modify pest distribution ranges. At the same time, the trade of living plants and fruits and human movements are increasing the risk of introduction of pests to new territories where they can spread and establish. This happens despite commodity and human movement regulations, and quarantine services and pest surveillance systems at border inspection points.

Tephritidae are one of the most speciose families in Diptera [1,2] with more than 4600 species [3]. The genus *Bactrocera* Macquart has more than 440 described species in the Old World including severe pests of fruit- and seed-bearing organs [4].

Several tephritids thrive in the native rainforest habitats of Southeast Asia and Australasia, with a high degree of host specialization and a large number of cryptic species [5,6].

The fruit flies belonging to the *Bactrocera dorsalis* complex and the species *Bactrocera zonata* (Saunders) are highly polyphagous and among the most alarming species. The pest status of *Bactrocera dorsalis* (Hendel) (the Oriental fruit fly) is considered higher than *B. zonata* (the peach fruit fly) and *Zeugodacus cucurbitae* (Coquillett) (the melon fly), with which it shares some host crops. Due to high polyphagy, high reproductive potential, an uninterrupted activity throughout most of the year and wide spreading ability, *B. dorsalis* represents the biggest threat to European fruit orchards.

In recent years, some fruit flies have expanded their distribution range invading new continents. Some populations of *B. dorsalis* have established on the Hawaiian Islands [7,8]. In March 2003, an invasive fruit fly species was detected in coastal parts of Kenya [9] and was described as *Bactrocera invadens* by Drew, Tsuruta and White [10]. However, it was subsequently synonymized with *B. dorsalis* [11,12,13,14].

At the same time, the Asian *Bactrocera zonata* has been recorded in Israel and North Africa, and it has already ecologically displaced *Ceratitis capitata* Wiedemann in Egypt [15,16]. Due to its potential economic impact, the invasion of this fly in the rest of the EPPO (European and Mediterranean Plant Protection Organization) area raises growing concerns.

Males of at least 34 species of the genus *Bactrocera* are attracted by the male lure methyl-eugenol (4-allyl-1, 2-dimethoxybenzene carboxylate) [5,17] and this male attractant is commonly used to monitor tephritids.

The aim of the present work was to determine the presence of specimens belonging to the *Bactrocera dorsalis* species complex in the Campania Region (Italy), following the European Directive 2000/29/EC of 8 May 2000 which states protective measures against the introduction into the community of organisms harmful to plants or plant products and against their spread within the community.

## 2. Materials and Methods

### 2.1. Fruit Fly Trapping

**Figure 1:**
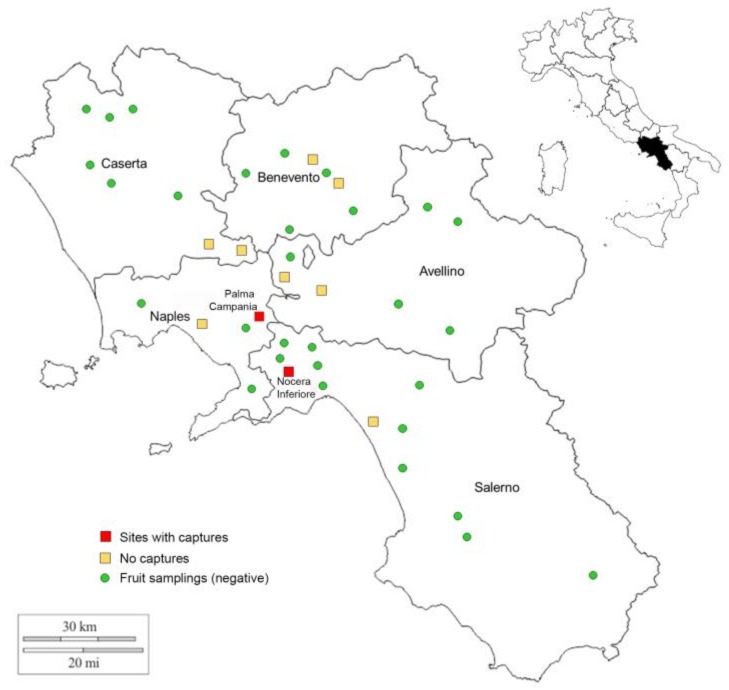
Monitoring of *Bactrocera dorsalis* in Campania Region during 2018 and related results. Squares are the locations where the traps were placed while green circles are fruit sampling sites.

In April and May 2018, ten traps baited with methyl-eugenol were placed in ten trapping locations (Figure 1), two fields for each province.

All sites were chosen for specific characteristics, such as a high variety of cultivated species, to ensure the presence of ripe fruits throughout the monitoring period. All fields were non-specialized orchards, having several fruit species together (*Citrus* spp., *Prunus* spp., *Vitis* sp., *Pyrus* spp., *Malus domestica*, and *Diospyros kaki*). Pest management strategies adopted in these orchards ranged from the total absence of chemical treatments (in the large part of chosen orchards) to integrated management. No fruit market is near the chosen orchards but there are some suburban gardens with several fruit trees and crops (*Solanum lycopersicum*, *Capsicum* spp., *Solanum melongena*, *Cucurbita* spp., *Citrus* spp., *Prunus* spp., etc.). In each site, one *McPhail* trap (Fly Catcher trap, CAT-F10^®^, Russel IPM, Deeside, UK) baited with methyl-eugenol (PH-136-1RR^®^, Russel IPM) was placed on trees at about 1.5 m above the ground level. A plug with the methyl-eugenol was suspended from the trap’s ceiling with a tie wire. Traps were checked weekly up to October 2018 and the male lure was changed every 6 weeks, as indicated by the Russel IPM.

Moreover, once a month a sampling of infested fruits was performed both in monitored fruit orchards and in other fields (Figure 1). Infested fruits were placed in sealed plastic bags in a refrigerator and carried to the laboratory, where they were arranged in plastic boxes in a climatic chamber (25 ± 2 °C, photoperiod 16L:8D, 60 ± 10% relative humidity) up to the pupation. Puparia were placed in glass vials (10 cm long, 1 cm ø, with a cotton cover) at the same temperature and humidity conditions and checked up to adult emergence. Emerged adults were identified as later reported. Adults collected with traps were placed singularly in a vial containing 90% ethanol and stored at −20 °C in the lab until use.

Since the first capture, in the fields where the first specimens were recorded, yellow sticky traps (Rebell^®^ Amarillo, Andermatt Biocontrol, Grossdietwil, Switzerland) baited with fed attractants (ammonium carbonate) were placed on trees at about 1.5 m above the ground level at least 20 m from the Fly Catcher trap.

### 2.2. Morphological Identification of Fruit Fly

The fruit flies captured in traps baited with methyl eugenol were collected and identified following the available taxonomic keys, insect descriptions, and comparative images [6,10,18,19,20,21,22].

An Axiocam HRC digital camera attached to a Zeiss Axiophot 2 microscope (Carl Zeiss, Oberkochen, Germany) was used to take photos. Multiple planes of focus were combined using CombineZP^®^ software to produce fully-focused images.

### 2.3. Molecular Characterization of Fruit Fly

From each specimen listed in Table 1, a leg (tibia and tarsus) was separated with sterile tweezers and scalpel blade and used for the total genomic DNA extraction. DNA extraction was performed using a Chelex and proteinase K based method as in Gebiola et al. [23] and pinned. After DNA extraction, each leg was rinsed with distilled water and preserved in absolute alcohol with the respective specimen. Hence, we sequenced the mitochondrial gene *cytochrome c oxidase* subunit I (COI). Primer pair LCO-1490/HCO-2198 [24] was used to amplify portions of ~640 bp (COI-A) with the thermocycler condition as in [23] and primer pair C1-J-2183/TL2-N-3014 [25], following the cycling conditions described in [26] to amplify ~810 portions (COI-B). Some authors highlighted the nuclear gene ITS1 was useful in the discrimination of *B. dorsalis* and *B. kandiensis* due to the absence and presence of an indel, respectively [13]. Hence, we sequenced ITS1 genes with the primers and PCR cycling conditions, as reported in [27]. PCR products were checked on a 1.2% agarose gel stained with GelRED^®^ (Biotium, Fremont, CA, USA) and directly sequenced. Chromatograms were assembled using BioEdit 7.0 [28] and edited manually.

COI sequences were virtually translated into amino acids to detect frameshift mutations and nonsense codons using EMBOSS Transeq (http://www.ebi.ac.uk/Tools/st/emboss_transeq/ (accessed 8 October 2018)).

COI and ITS1 sequences were blasted against the homologous sequences available in GenBank database (last access 8 October 2018 and 7 November 2018, respectively); furthermore, COI sequences were matched against BOLD database also through BOLD Identification System (www.boldsystems.org, last access 5 November 2018).

Obtained sequences were deposited in GenBank with accession numbers reported in Table 1.

Due to the absence of a satisfactory number of complete COI sequences in the Genbank database (last access 8 October 2018), essential for the taxonomic placement of the studied samples, the obtained portions were separately investigated. Alignments of COI-A portions were assembled with sequences acquired in [13], sorted on the basis of place and collection date, and identical sequences were excluded, as suggested by RAxML 7.0.4 [29]. COI-B portions were aligned with corresponding sequences of members of *B. dorsalis* complex gained in [30].

With the aim to identify the collected specimens with the highest degree of accuracy, the phylogenies on COI alignments were reconstructed using maximum likelihood (ML) with RAxML 7.0.4 [29], implementing for both alignments the GTR+G+I evolutionary models as selected by jModeltest [31]. ML branch support was based on 10,000 rapid bootstrap pseudoreplicates and clades were considered supported when bootstrap was >70%. *Bactrocera tryoni* (Froggatt) was used as the outgroup to root the COI-A tree, while the COI-B tree was rooted using the midpoint-rooted tree option.

### 2.4. Morphological Re-Examination

The last step of the iterative identification process was a re-examination of the systematic characters that morphologically distinguish our samples from the closer species according to the mtDNA results.

## 3. Results

### 3.1. Fruit Fly Trapping

During 10–30 September 2018, traps baited with methyl-eugenol caught seven male specimens of fruit fly in two different localities (Table 1).

Both traps that captured males were placed in non-specialized orchards, as reported in Materials and Methods. At that time, there were unripe fruits of persimmon and citrus (oranges and clementines) and some residual fruit of plums in Nocera Inferiore. In Palma Campania, there were the same plants and the same fruits with some bunches of grapes. Near both fields, there were tomato plants with ripe fruits and some pumpkin plants and fruits. In Nocera Inferiore, an integrated pest management strategy is adopted, while, in Palma Campania, no chemical treatments are performed.

Traps baited with fed attractants, placed after the first fly records, caught only *Bactrocera oleae* (Rossi) among other common fruit fly species.

From puparia collected from sampled infested fruits (mainly citrus and persimmon), only *C. capitata* adults emerged.

### 3.2. Morphological Identification of Fruit Fly

**Figure 2:**
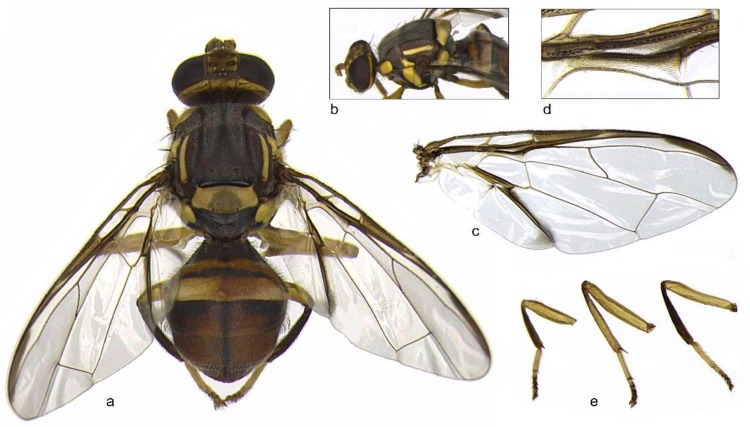
*Bactrocera dorsalis* male (specimen BD_2): dorsal view (**a**); lateral view with particular of the postpronotal lobe and lateral vitta (**b**); right wing (**c**); microtrichia pattern in cell *br* (**d**); and fore, mid, and hind legs (**e**).

All fruit fly specimens caught with traps baited with methyl-eugenol were identified as belonging to *B. dorsalis* (Eppo code: DACUDO) species in *B. dorsalis* complex (Figure 2).

### 3.3. Molecular Characterization of Fruit Fly

Identical COI haplotypes were obtained from specimens recorded in Nocera Inferiore (BD_1 and BD_2), which resulted different from COI haplotype of the specimen collected in Palma Campania (BD_3).

BLAST searches with the amplified portions of COI-A and COI-B of specimens from Nocera Inferiore resulted in a similarity of 99% with some sequences of *B. dorsalis* and several sequences of *B. kandiensis*. COI-A sequence from BD_3 resulted 99% similar to several sequences of *B. dorsalis*, *B. carambolae* Drew & Hancock and *B. papayae* (*B. dorsalis* syn., [13]). Analogous results were recorded for the COI-B portion, where, however, the preponderance of *B. dorsalis* was highlighted.

Matching against BOLD database resulted in uncertain identifications at the species level. Indeed, 100% similarity was found with unspecified *Bactrocera* samples (sequences not already published), while a similarity ranging from 99.84% to 99.67% was found with several *Bactrocera* spp. (*B. dorsalis*, *B. papayae*, *B. carambolae*, *B. kandiensis*, *B. zonata*, *B. verbascifoliae* Drew & Hancock, *B. invadens*, and *B. irvingiae* Drew & Hancock).

**Figure 3:**
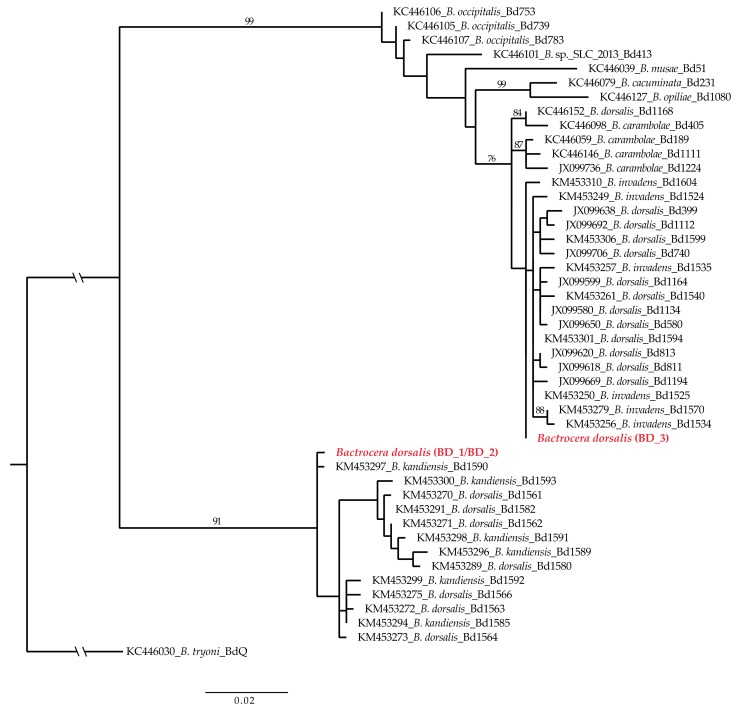
Maximum likelihood consensus tree for the mitochondrial portion dataset COI-A of the *Bactrocera* species selected from Schutze et al. [13]. Bootstrap values of >70% are shown above branches.

Phylogenetic analysis of the COI-A (Figure 3) showed specimens BD_1 and BD_2 are very close to *B. kandiensis*, clustering in the same supported clade along with several *B. dorsalis* specimens from Sri Lanka, Myanmar, and India. BD_3 specimen instead fell close to a broader group that includes *B. dorsalis*, *B. invadens* (*B. dorsalis* syn.), and *B. carambolae*, but without inclusion in a clade or a specific subclade. COI-B phylogenetic tree (Figure S1) showed BD_1 and BD_2 are closely related to *B. kandiensis* while BD_3 grouped with *B. carambolae*, *B. dorsalis* 4345 [initial identification (I. i.): *B. dorsalis*], *B. dorsalis* 1432 (I. i: *B. papayae*), and *B. dorsalis* 3471 (I. i.: *B. invadens*).

BD_2 and BD_3 share identical ITS1 sequence while BD_1 showed a 3 bp deletion. Blast searches with the ITS1 sequences for samples BD_2 and BD_3 resulted in a 100% similarity with *B. dorsalis* sequence (voucher bd1583—accession number KM453381) while BD_1 showed 100% similarity with *B. dorsalis* sequence (voucher bd1697—accession number KM453414).

### 3.4. Morphological Re-Examination

Based on molecular results, our specimens resulted morphologically different from *B. kandiensis* because:

(a) They have completely yellow femora rather than have a black spot on each femur as *B. kandiensis (*Figure 2e) [6].
(b) The microtrichia pattern in cell *br* is different from that reported for *B. kandiensis * that has a bare colorless area adjacent to cell *bm* approximately one half the length of this cell as in *B. dorsalis* (Figure 2d) [10,18].
(c) The black spot on the postpronotal lobe that should easily separate *B. kandiensis * from the other fruit fly species of *B. dorsalis* complex [18] is missing (Figure 2b).
(d) The abdominal terga III–V have a moderately broad medial longitudinal dark band and broad lateral longitudinal dark bands [20].

## 4. Discussion

The number of invasive species that have been found in Italy in recent years is very high [32,33,34,35,36,37]. This is probably due to its climate and geographical position, which allow the settlement of exotic species.

Researchers working on the *B. dorsalis* complex know that an indisputable identification of taxa is tangled, even though almost all species are included within a unique taxonomic key (excluding the Australian fauna) [20]. However, following the existing keys, the specimens collected in Italy were identified as *B. dorsalis*, although our integrative approach aimed at characterizing collected specimens showed an apparent incongruence because morphological approach clearly identified specimens caught in traps baited with methyl-eugenol as belonging to *B. dorsalis* while molecular approach showed that specimens collected in Nocera Inferiore are very close to *B. kandiensis* for COI. However, both the sequencing of ITS1 (the lack of indel present in *B. kandiensis* sequences [13]) and the morphological re-examination (at least four distinct morphological characters) confirmed that the trapped specimens do not belong to *B. kandiensis* species.

A similar incongruence between morphological and some molecular results has been previously recorded with some specimens of *B. dorsalis* that resulted to have COI haplotypes more closely related to *B. kandiensis* than other *B. dorsalis* haplotypes [13]. The presence of *B. kandiensis* haplotypes among *B. dorsalis* individuals could be due to introgression via hybridization [38].

Contrary to the incongruent results obtained with BD_1 and BD_2 samples, both morphological and molecular characterizations of the specimen caught in Palma Campania (BD_3) coincided. The apparent incongruence of our results is probably due to the great systematic uncertainty that reigns in this complex of species, where some species may not be distinguishable with confidence by means other than DNA data [14]. Moreover, recent results showed *B. dorsalis* complex is a polyphyletic assemblage [30].

The high phenotypic plasticity of the species of this group causes a high intraspecific color and morphometric polymorphism [14,39] and therefore an overlap of the variation range of some crucial systematic characters. This may also cause incorrect morphological identifications that may have brought to the submissions in Genbank and BOLD databases of sequences referring to misidentified species.

Since 1995, *B. dorsalis* complex (including *B. kandiensis)* was in the EPPO A1 list of pests recommended for regulation as quarantine pests and in the EU Annex II/A1: “*Pests known not to occur in the EU, whose introduction into, and/or whose spread within the EU is prohibited due to their dangerousness*”. However, *B. kandiensis* is ranked in category B of severity based on its limited distribution range [40,41]. *B. kandiensis* is highly polyphagous and has been recorded on several, unrelated, host plant families [6,42]. Similarly, *B. dorsalis* has been recorded on a large number of different host fruits and vegetables (more than 250 species) [43] and an exhaustive host list is provided in [44]. Both *B. dorsalis* and *B. kandiensis* are considered to have economically important host plants and both species can be monitored using methyl eugenol.

*Bactrocera dorsalis* is widespread (Asia, Africa, Oceania, and Hawaiian Islands), while *B. kandiensis* is endemic to Sri Lanka and sympatric with *B. invadens*, which recently was synonymized with *B. dorsalis* [11,12,13,14]. Both *B. dorsalis* and *B. kandiensis* are members of the *B. dorsalis* complex, are pest species in their own right, and are morphologically similar [6,43,45]. This resemblance has often resulted in some confusion as to its biological relationship with *B. dorsalis.* However, *B. kandiensis* possesses subtle yet consistent differences in morphology [6,46,47] and molecular genetics [11,13,27] sufficient to regard it as distinct species [11].

According to the Europhyt database (EU Member states only), several interceptions of *B. dorsalis* in Europe are reported annually in France, Switzerland, and the UK in consignments of infested fruits and vegetables [48]. However, to date, only a single trapping detection of a single specimen of *B. dorsalis* in a fruit market in Austria in 2016 is published [49,50].

The findings presented here are the first in open field targeting orchard environment in Europe.

The winter temperatures in Italy could be too low for the survival of both *B. kandiensis*, which is native to a tropical country (Sri Lanka), and *B. dorsalis* [51]. However, *B. dorsalis* is continually spreading in central and northern China, thus suggesting its overwintering ability in similar climatic zones to the temperate regions of Europe [52] and ability to acclimate.

The absence of *B. dorsalis* from countries close to Italy implies that it has arrived through the introduction of infested fruits or adults because, even if some species belonging to *Bactrocera* genus are able to fly for 50–100 km [44], no infested country is so close.

In recent years, during our activity in the support of customs phytosanitary service, the finding of small quantities of mango and other unregulated tropical fruits in passengers’ luggage at the airport has undergone a considerable increase and it is possible that this was the route followed by the fruit fly to enter Italy [53].

The current distribution of *B. dorsalis* in Campania seems to be still limited both because only two out of ten placed traps captured some specimens and because the sampled infested fruits resulted to be infested exclusively by *C. capitata*.

Another clue that makes us assume the temporal proximity of the introduction is the evidence of no record of *B. dorsalis* in the last five years. Indeed, in the same orchard in Palma Campania (where a male was recorded), we carried out a monitoring survey for *Rhagoletis completa* Cresson using 12 traps baited with fed attractant (that is mildly attractive to *B. dorsalis* [54]). In our previous trials, no specimens of *B. dorsalis* complex were collected (Bernardo et al. in prep.). The finding of two phylogenetically distant haplotypes could suggest that the infestation started due to at least two independent introductions. Moreover, we cannot reconstruct the invasive pathway process of the introduced population because the specimens caught in Italy showed different haplotypes than homologous sequences present in Genbank, while the homologous sequences present in the BOLD SYSTEMS refer to unidentified species of the genus *Bactrocera* (*Bactrocera* sp.) despite the many sequences of *B. dorsalis* and therefore the many different haplotypes present in the database. A population genetic study using microsatellite markers could be the next step of our research to trace the invasive pathways of the Italian population of *B. dorsalis* [55].

In the near future, we plan to extend our activities of monitoring by direct fruit sampling and increasing the use traps baited with both methyl-eugenol and fed attractants (ammonium carbonate), and to also evaluate if the present population will be able to overwinter.

## 5. Conclusions

This is the first record of *B. dorsalis* specimens in fruit orchards in Europe; this finding can strongly affect both the production in Italian orchards and crops and the commercial exchanges of Italian fruits in Europe due to the existing quarantine measures.

The genetic differences between the haplotypes could suggest that at least two different introductions took place. The two infested sites are 15 km away from each other but other infested fields are not to be excluded in the surrounding areas, especially due to the highly agricultural predominance of the landscape.

In the next year, the monitoring activities will be strongly increased (also involving neighboring Italian regions) and will be aimed at ascertaining the presence of other infested fields, verifying the occurrence of overwintering phenomena and monitoring the fruit damage in the area.

## Figures and Tables

**Table 1:** Number of males collected in traps baited with methyl-eugenol grouped according to the locality of sampling. The numbers in brackets indicate the specimens whose COI and ITS1 were sequenced.

Voucher	Localities	N. of Caught Flies	Province	Coordinates	Genbank Accession Code		
					LCO-HCO	2183-3014	ITS1
BD_1	Nocera Inferiore	6(2)	Salerno	40°44′ N, 14°37′ E	MK106007	MK106010	MK158099
BD_2					MK106008	MK106011	MK158100
BD_3	Palma Campania	1(1)	Naples	40°51′ N, 14°33′ E	MK106009	MK106012	MK158101

## Supplementary Materials

The following are available online at http://www.mdpi.com/2075-4450/9/4/182/s1, Figure S1: Maximum likelihood consensus tree for the mitochondrial portion dataset COI-B tree of the species of *B. dorsalis* complex. Bootstrap values >70% are shown above branches.
